# Supplementary material for: KATANIN-dependent mechanical properties of the stigmatic cell wall mediate the pollen tube path in Arabidopsis
Source: eLife. 2020 Sep 1;9:e57282. doi: 10.7554/eLife.57282 (PMC7462616; doi:10.7554/eLife.57282)
Supplement: Figure 5—source data 2. [file elife-57282-fig5-data2.docx]

**Figure 5–source data 2. Table of cell wall mutants analysed**

| Cell wall component | AGI/Name | Function* | Mutant | References | Expression in stigma** |
| --- | --- | --- | --- | --- | --- |
| Cellulose | AT5G49720 *KOR1* | Endoglucanase 25 | *kor1* | His et al., 2001; Lei et al., 2014; Nicol et al., 1998 | Yes |
| Cellulose | AT5G64740 *PRC1* | Cellulose synthaseA catalytic subunit 6 | *prc1* | Fagard et al., 2000; MacKinnon et al., 2006; Panteris et al., 2014; Xiao et al., 2016 | Yes |
| Cellulose | AT4G32410 *ANY1* | Cellulose synthaseA catalytic subunit 1 | *any1* | Fujita et al., 2013 | Yes |
| Hemicellulose | AT3G62720 *XXT1*  AT4G02500 *XXT2* | Xyloglucan 6-xylosyltransferase 1  Xyloglucan 6-xylosyltransferase 2 | *xxt1 xxt2* | Cavalier et al., 2008; Xiao et al., 2016 | Yes |
| Hemicellulose | AT1G68560 *XYL1* | Alpha-xylosidase 1 | *xyl1.4* | Sechet et al., 2016 | Yes |
| Pectin | AT1G78240 *QUA2* | Probable Pectin methyltransferase | *qua2.1* | Abasolo et al., 2009; Mouille et al., 2007; Verger et al., 2018 | Yes |

*From <https://www.uniprot.org>

**From Kodera et al., 2018 (<https://doi.org/10.1101/374843>) and ePlant: <https://bar.utoronto.ca/eplant/>

Abasolo W, Eder M, Yamauchi K, Obel N, Reinecke A, Neumetzler L, Dunlop JWC, Mouille G, Pauly M, Höfte H, Burgert I. 2009. Pectin May Hinder the Unfolding of Xyloglucan Chains during Cell Deformation: Implications of the Mechanical Performance of Arabidopsis Hypocotyls with Pectin Alterations. *Molecular Plant* **2**:990–999. doi:10.1093/mp/ssp065

Cavalier DM, Lerouxel O, Neumetzler L, Yamauchi K, Reinecke A, Freshour G, Zabotina OA, Hahn MG, Burgert I, Pauly M, Raikhel NV, Keegstra K. 2008. Disrupting Two Arabidopsis thaliana Xylosyltransferase Genes Results in Plants Deficient in Xyloglucan, a Major Primary Cell Wall Component. *Plant Cell* **20**:1519–1537. doi:10.1105/tpc.108.059873

Fagard M, Desnos T, Desprez T, Goubet F, Refregier G, Mouille G, McCann M, Rayon C, Vernhettes S, Höfte H. 2000. PROCUSTE1 Encodes a Cellulose Synthase Required for Normal Cell Elongation Specifically in Roots and Dark-Grown Hypocotyls of Arabidopsis. *Plant Cell* **12**:2409–2423. doi:10.1105/tpc.12.12.2409

Fujita M, Himmelspach R, Ward J, Whittington A, Hasenbein N, Liu C, Truong TT, Galway ME, Mansfield SD, Hocart CH, Wasteneys GO. 2013. The anisotropy1 D604N mutation in the Arabidopsis cellulose synthase1 catalytic domain reduces cell wall crystallinity and the velocity of cellulose synthase complexes. *Plant Physiol* **162**:74–85. doi:10.1104/pp.112.211565

His I, Driouich A, Nicol F, Jauneau A, Höfte H. 2001. Altered pectin composition in primary cell walls of korrigan, a dwarf mutant of Arabidopsis deficient in a membrane-bound endo-1,4-beta-glucanase. *Planta* **212**:348–358. doi:10.1007/s004250000437

Lei L, Zhang T, Strasser R, Lee CM, Gonneau M, Mach L, Vernhettes S, Kim SH, J. Cosgrove D, Li S, Gu Y. 2014. The *jiaoyao1* Mutant Is an Allele of *korrigan1* That Abolishes Endoglucanase Activity and Affects the Organization of Both Cellulose Microfibrils and Microtubules in Arabidopsis. *Plant Cell* **26**:2601–2616. doi:10.1105/tpc.114.126193

MacKinnon IM, Šturcová A, Sugimoto-Shirasu K, His I, McCann MC, Jarvis MC. 2006. Cell-wall structure and anisotropy in *procuste*, a cellulose synthase mutant of *Arabidopsis* *thaliana*. *Planta* **224**:438–448. doi:10.1007/s00425-005-0208-6

Mouille G, Ralet M-C, Cavelier C, Eland C, Effroy D, Hématy K, McCartney L, Truong HN, Gaudon V, Thibault J-F, Marchant A, Höfte H. 2007. Homogalacturonan synthesis in *Arabidopsis thaliana* requires a Golgi‐localized protein with a putative methyltransferase domain. *Plant Journal* **50**:605–614. doi:10.1111/j.1365-313X.2007.03086.x

Nicol F, His I, Jauneau A, Vernhettes S, Canut H, Höfte H. 1998. A plasma membrane-bound putative endo-1,4-beta-D-glucanase is required for normal wall assembly and cell elongation in Arabidopsis. *EMBO J* **17**:5563–5576. doi:10.1093/emboj/17.19.5563

Panteris E, Adamakis I-DS, Daras G, Rigas S. 2014. Cortical microtubule patterning in roots of *Arabidopsis thaliana* primary cell wall mutants reveals the bidirectional interplay with cell expansion. *Plant Signal Behav* **9**. doi:10.4161/psb.28737

Sechet J, Frey A, Effroy-Cuzzi D, Berger A, Perreau F, Cueff G, Charif D, Rajjou L, Mouille G, North HM, Marion-Poll A. 2016. Xyloglucan metabolism differentially impacts cell wall characteristics of the endosperm and embryo during Arabidopsis seed germination. *Plant Physiol* **170**:1367–1380. doi10.1104/pp.15.01312

Verger S, Long Y, Boudaoud A, Hamant O. 2018. A tension-adhesion feedback loop in plant epidermis. *eLife*. doi:10.7554/eLife.34460

Xiao C, Zhang T, Zheng Y, Cosgrove DJ, Anderson CT. 2016. Xyloglucan Deficiency Disrupts Microtubule Stability and Cellulose Biosynthesis in Arabidopsis, Altering Cell Growth and Morphogenesis. *Plant Physiol* **170**:234–249. doi:10.1104/pp.15.01395
