# Supplementary material for: KATANIN-dependent mechanical properties of the stigmatic cell wall mediate the pollen tube path in Arabidopsis
Source: eLife. 2020 Sep 1;9:e57282. doi: 10.7554/eLife.57282 (PMC7462616; doi:10.7554/eLife.57282)
Supplement: Transparent reporting form [file elife-57282-transrepform.pdf]

1st Floor
24 Hills Road
Cambridge CB2 1JP, UK

P 01223 855340
W elifesciences.org
T @elife

eLife's transparent reporting form

We encourage authors to provide detailed information *within their submission* to facilitate the interpretation and replication of experiments. Authors can upload supporting documentation to indicate the use of appropriate reporting guidelines for health-related research (see EQUATOR Network), life science research (see the BioSharing Information Resource), or the ARRIVE guidelines for reporting work involving animal research. Where applicable, authors should refer to any relevant reporting standards documents in this form.

If you have any questions, please consult our Journal Policies and/or contact us: editorial@elifesciences.org.

Sample-size estimation

- You should state whether an appropriate sample size was computed when the study was being designed
- You should state the statistical method of sample size computation and any required assumptions
- If no explicit power analysis was used, you should describe how you decided what sample (replicate) size (number) to use

Please outline where this information can be found within the submission (e.g., sections or figure legends), or explain why this information doesn't apply to your submission:

- For each experiment, sample size and relevant statistical tests have been specified in the figure legends.

Replicates

- You should report how often each experiment was performed
- You should include a definition of biological versus technical replication
- The data obtained should be provided and sufficient information should be provided to indicate the number of independent biological and/or technical replicates
- If you encountered any outliers, you should describe how these were handled
- Criteria for exclusion/inclusion of data should be clearly stated
- High-throughput sequence data should be uploaded before submission, with a private link for reviewers provided (these are available from both GEO and ArrayExpress)

Please outline where this information can be found within the submission (e.g., sections or figure legends), or explain why this information doesn't apply to your submission:

eLife Sciences Publications, Ltd is a limited liability non-profit non-stock corporation incorporated in the State of Delaware, USA, with company number 5030732, and is registered in the UK with company number FC030576 and branch number BR015634 at the address 1st Floor, 24 Hills Road, Cambridge CB2 1JP | August 2014

- Each experiment was performed with at least 3 independent biological samples (i.e biological replicates). Number of samples analysed is given in the figure legends and in source data.

For the chemical composition of cell walls, Figure 5-figure supplement 4, the monosaccharide composition of the cell wall for all replicates and genotypes was performed the same day in strictly identical conditions (same conditions for hydrolysis, same heat block, and one single run for HPAEC-PAD detection of sugars). 30 to 50 pistils from different plants of one genotype were pooled to form 1 replicate. See Materials and methods.
Similarly, the xyloglucan fingerprint was performed the same day in strictly identical conditions (all digestions at the same time, same enzyme solution, same heat block and one single preparation for MALDI-TOF analysed at the same time). 10 dissected stigmas of one genotype were pooled to obtain 1 biological replicate. See Materials and methods.

Statistical reporting

- Statistical analysis methods should be described and justified
- Raw data should be presented in figures whenever informative to do so (typically when N per group is less than 10)
- For each experiment, you should identify the statistical tests used, exact values of N, definitions of center, methods of multiple test correction, and dispersion and precision measures (e.g., mean, median, SD, SEM, confidence intervals; and, for the major substantive results, a measure of effect size (e.g., Pearson's r, Cohen's d)
- Report exact p-values wherever possible alongside the summary statistics and 95% confidence intervals. These should be reported for all key questions and not only when the p-value is less than 0.05.

Please outline where this information can be found within the submission (e.g., sections or figure legends), or explain why this information doesn't apply to your submission:

- Statistical tests used for each experiment can be found in the legend of the figures, with the sample size, the standard error of the mean and the p-value.
- Blinding were used to calculate the deformation made by the pollen tube in Figure 6D. For all the cell wall analyses, standard deviations were calculated with the 3 replicates.

(For large datasets, or papers with a very large number of statistical tests, you may upload a single table file with tests, Ns, etc., with reference to sections in the manuscript.)

Group allocation

- Indicate how samples were allocated into experimental groups (in the case of clinical studies, please specify allocation to treatment method); if randomization was used, please also state if restricted randomization was applied
- Indicate if masking was used during group allocation, data collection and/or data analysis

eLife Sciences Publications, Ltd is a limited liability non-profit non-stock corporation incorporated in the State of Delaware, USA, with company number 5030732, and is registered in the UK with company number FC030576 and branch number BR015634 at the address 1st Floor, 24 Hills Road, Cambridge CB2 1JP | August 2014

Please outline where this information can be found within the submission (e.g., sections or figure legends), or explain why this information doesn't apply to your submission:

In this study, groups such as mutant vs wild-type or mock vs treated were used, as described in the figure legends or directly in the main text.

Additional data files ("source data")

- We encourage you to upload relevant additional data files, such as numerical data that are represented as a graph in a figure, or as a summary table
- Where provided, these should be in the most useful format, and they can be uploaded as "Source data" files linked to a main figure or table
- Include model definition files including the full list of parameters used
- Include code used for data analysis (e.g., R, MatLab)
- Avoid stating that data files are "available upon request"

Please indicate the figures or tables for which source data files have been provided:

Source data are provided for Figure 1, 2, 4, 5, 6 and Figure 5-figure supplement 2, 3 and 4.
